# Grade C Molar-Incisor Pattern Periodontitis in Young Adults: What Have We Learned So Far?

**DOI:** 10.3390/pathogens13070580

**Published:** 2024-07-12

**Authors:** Manuela Maria Viana Miguel, Luciana Macchion Shaddox

**Affiliations:** 1 Center for Oral Health Research, College of Dentistry, University of Kentucky, Lexington, KY 40508, USA; vianamiguel.manuela@gmail.com; 2 Department of Oral Health Practice, Periodontology Division, College of Dentistry, University of Kentucky, Lexington, KY 40508, USA

**Keywords:** periodontitis, aggressive, immunological factors, microbiota, genetics

## Abstract

Grade C molar-incisor pattern periodontitis (C-MIP) is a disease that affects specific teeth with an early onset and aggressive progression. It occurs in systemically healthy patients, mostly African descendants, at an early age, with familial involvement, minimal biofilm accumulation, and minor inflammation. Severe and rapidly progressive bone loss is observed around the first molars and incisors. This clinical condition has been usually diagnosed in children and young adults with permanent dentition under 30 years of age. However, this disease can also affect the primary dentition, which is not as frequently discussed in the literature. Radiographic records have shown that most patients diagnosed in the permanent dentition already presented disease signs in the primary dentition. A hyperresponsive immunological profile is observed in local (gingival crevicular fluid-GCF) and systemic environments. Siblings have also displayed a heightened inflammatory profile even without clinical signs of disease. *A. actinomycetemcomitans* has been classified as a key pathogen in C-MIP in both dentitions. Scaling and root planning associated with systemic antibiotics is the current gold standard to treat C-MIP, leading to GCF biomarker reduction, some systemic inflammatory response modulation and microbiome profile changes to a healthy-site profile. Further studies should focus on other possible disease-contributing risk factors.

## 1. Clinical Overview

**Figure 1:**
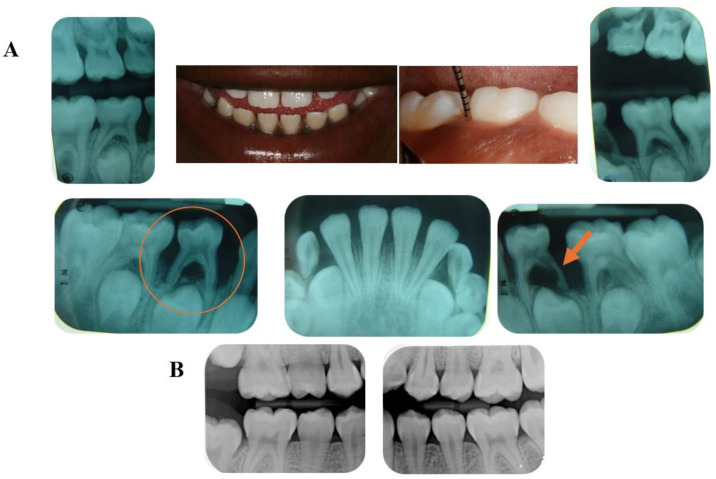
Clinical Case: 8-year-old African American female diagnosed with C-MIP in primary dentition. Patient smiles with low gingival margin inflammation; however, probing depth > 5 mm in the first molar. In the X-ray, severe bone loss in the lower first primary molars (orange arrow and circle) along with internal and external resorption of the lower left primary first molar in the primary dentition (orange arrow) (**A**). Permanent dentition in healthy conditions following therapy (SRP+ABX) in the primary dentition (**B**). (Source of the image [10]).

Periodontitis is a biofilm-dependent condition characterized by a shift in the resident microbiota, leading to increased host response to its challenge [1]. As a result of this dysbiosis and host immunological profile, destruction of the tooth-supporting apparatus [2] can clinically be detected in different progression and severity patterns. According to the 1999 classification of periodontal diseases and conditions [3], Localized Aggressive Periodontitis (LAP) was characterized to occur in systemically healthy patients at an early age with familial involvement, minimal biofilm accumulation, and low gingival margin inflammation, severe bone loss along with rapid clinical attachment loss (CAL) progression, specifically in first molars and incisors. According to the new periodontitis classification based on stages and grades, LAP was then named Grade C molar-incisor pattern periodontitis (C-MIP) [4]. Epidemiological data have provided evidence that early onset aggressive periodontal condition oscillates in different populations, where higher prevalences can be observed in African descendants (up to 6% of prevalence) and the lowest in Caucasians (less than 1%) [5,6]. Along with the African continent, South America comprises a prevalence of 0.3–5% of the disease [5,7] which may be correlated to locations with a mixed-race population. This pattern suggests a specific geographic distribution of the disease. This clinical condition has been frequently diagnosed in older children and young adults in permanent dentition under 30–35 years of age [6,8]. However, it is important to highlight that primary dentition can also be affected by Grade C Periodontitis [9,10,11,12]. The lack of periodontal assessment in pediatric dental examination may overlook this disease’s initial development, jeopardizing the actual timing of onset, and may contribute to the development of the disease in the permanent dentition, as suggested before [13,14,15,16]. In general, Grade C periodontitis in primary dentition can mostly be detected in primary molars and may be diagnosed late at severe stages or by early exfoliation due to disproportional bone loss occurring around these teeth in relation to the rate of physiological apical root resorption [10,16]. In addition, external and internal root resorptions in primary dentition are conditions that have also been reported in this disease [10,16] (Figure 1A). Early diagnosis in primary dentition displays a pivotal role in treatment success, along with strict periodontal maintenance follow-up to prevent the development of the disease in the permanent dentition (Figure 1B) [9,15,16,17]. Gender influence in LAP/C-MIP prevalence has been studied; however, inconsistent evidence has been reported [18]. Literature has shown similar [19] or higher [20] disease prevalence in females. However, due to some differences found in the inflammatory response in this disease between sexes [18], sex hormones may be the key to a better understanding of how gender may impact this disease (see further discussion below regarding host response).

## 2. Immunological Aspects 

**Figure 2:**
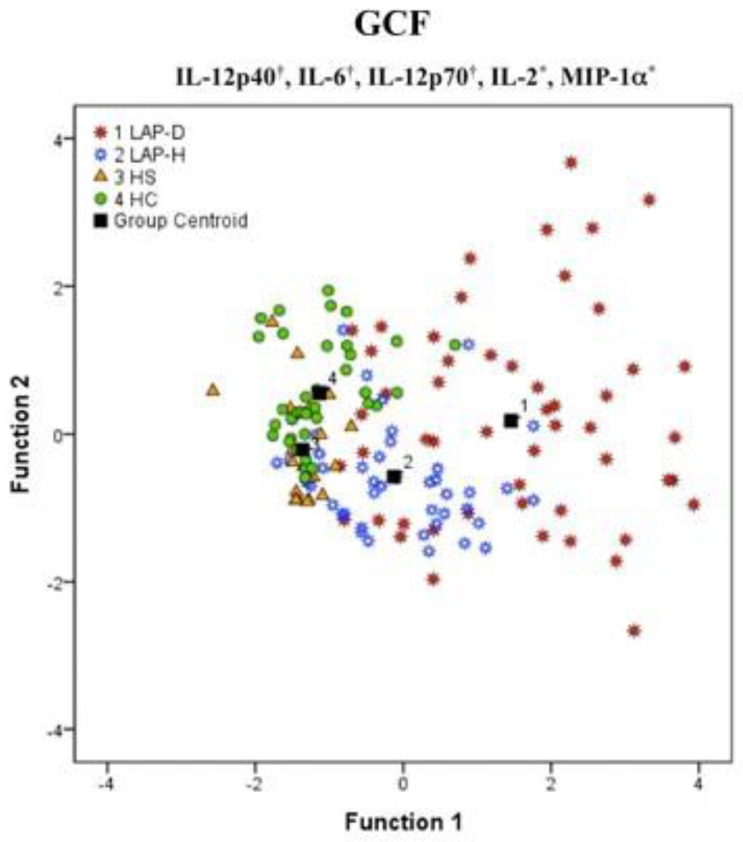
Discriminatory gingival crevicular fluid (GCF) in localized aggressive periodontitis (LAP) subjects, their healthy siblings (HS), and unrelated healthy controls (HC) displaying four different group centroids (1−4). The separation between LAP diseased sites (LAP−D) and LAP healthy sites (LAP−H) is clearly observed in the graphic along with a closely related profile (although statistically different) for HS sites and HC sites by a biomarker group of IL−12p−40, IL−6, IL−12p70, IL−2, and MIP−1α (Wilks’s lambda < 0.001 in canonical functions 1 and 2). * Significant biomarker in function 1. ^†^ Significant biomarker in function 2. (Source of the image [21]).

**Local inflammation.** Focusing on host immunological response complexity to a low biofilm aggregation, Branco-de-Almeida et al. [21] and Shaddox et al. [22] reported high levels of IL-12p40, GMCSF, TNF-α, IL-6, IL-12p70, IL-2, INFγ, IL1β, and MIP-1α gingival crevicular fluid (GCF) of LAP patients diseased sites when compared to health sites from the same patients [22] and/or health siblings and healthy unrelated controls [21,22] Interestingly, a significant differential profile of these markers was observed among these sites, where most differences fall between diseased and healthy sites, however, the healthy sites from healthy individuals tended to cluster more closely together (Figure 2). For instance, higher GCF concentrations of Eotaxin, GM-CSF, IL-10, IL-12p70, and IL-2 were observed in health sites from LAP patients when compared to their health siblings [21]. Additionally, healthy siblings of LAP subjects displayed an increase in IFN-γ and OPG without any clinical evidence of disease [21]. Interestingly, Martins et al. [23] showed similarity of some biomarker’s expression (IL1β, ICAM-1, and GMCSF) either in C-MIP or generalized aggressive forms of periodontitis (GAgP; CAL > 4 mm affecting at least three permanent teeth other than the first molars and incisors), at baseline to health controls. A current systematic review and meta-analysis [24] confirm that most of the cited biomarkers above were highly expressed in the GCF of C-MIP patients; however, the literature still needs more evidence to use this approach as a diagnosis tool. Some of these biomarkers have a pivotal role in stimulating cells’ environment towards osteoclastic activity and sustaining proinflammatory profile by chemotaxis. Thus, these differentiated local levels of inflammatory markers may contribute to local microbial dysbiosis and possible site breakdown in these individuals (see microbial discussion below).

**Figure 3:**
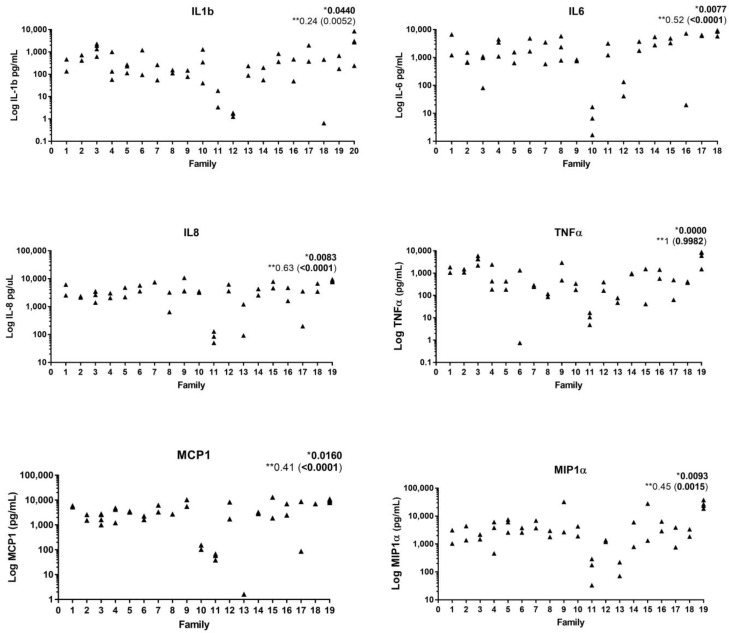
Significant correlations of host response to bacterial lipopolysaccharides within and among families with C-MIP. * Overall covariance *p*-value among families; ** Within families intraclass correlation coefficient followed by within family *p*-value in parenthesis. (Source of the image [27]).

**Systemic inflammation.** Peripheral blood from C-MIP stimulated either with LPS or biofilm from healthy and diseased sites has shown a hyperinflammatory responsiveness profile with increased levels of G-CSF, IFNγ, IL-10, IL12(p40), IL1β, IL-6, IL-8, MCP-1, MIP-1α, and TNFα when compared healthy control patients [25,26]. Interestingly, the similar response, regardless of the specific biofilm used for stimulation, highlights the role of host response in this unique disease. Tabaa et al. [27] have shown a similar pattern of proinflammatory response (IL-6, IL-8, IL-10, MIP1α, MCP1, and IL-1β) among affected siblings after peripheral blood LPS stimulation (Figure 3). Similarly, both *E. coli* and *P. gingivalis* LPS-stimulated peripheral blood from healthy siblings of LAP subjects has shown a tendency of hyperresponsiveness with IL2, IL12p40, TNFα, IFNγ, and IL6 elevated levels compared to unrelated health controls [25]. In fact, healthy siblings of LAP individuals tend to show a high peripheral response to diseased biofilms, showing increased levels of IL-6 and INFγ. In fact, even in response to healthy biofilm stimulation, this group had a higher concentration of INFγ compared to unrelated healthy patients [26]. This evidence collectively can point to two reasonable hypotheses. First, there seems to be a familial aggregation associated with this hyper-inflammatory response to bacterial stimuli in families with this disease [27]. Second, it can be hypothesized that inflammatory response could potentially precede or exacerbate bacterial dysbiosis in susceptible individuals in this clinical condition since both healthy and pathogenic biofilms have been shown to elicit a hyper-inflammatory host response in these individuals [26]. In addition, healthy sites in diseased patients display biomarkers featuring a differentiated and proinflammatory local profile [26]. 

The first line of defense in individuals who suffer from an aggressive form of periodontitis can be compromised due to either neutrophil hyperactivity [28] or dysfunction [29,30]. Van Dyke et al. [29] showed a deficiency in neutrophil chemotaxis in 26 out of 32 patients diagnosed with C-MIP/LAP compared to age and sex-matched healthy controls. This pattern remains even in those whose disease has already been treated. According to the same study, 7 out of 10 patients displayed neutrophil chemotaxis deficiency after 6 months to 2 years post-therapy [29]. Thus, it seems that a genetic determination may be associated with this neutrophil role. Van Dyke et al. [30] have shown neutrophil dysfunction in 90% of healthy siblings under 12 years old featuring a familial aggregation. This deficiency can be considered a predictor of aggressive forms of periodontitis since some of the healthy siblings detected with neutrophils at the beginning of the mentioned studies developed the disease during the study [30]. Focusing on humoral response, high levels of immunoglobulin G2 (IgG2 antibody) have also been observed in LAP patients [31]. Studies are controversial if this immunological response is specific to *A. actinomycetemcomitans* [32,33]; however, a racial influence was observed in this immunoglobulin levels [34], where higher concentrations of IgG2 serum was observed in African American descendants diagnosed with LAP when compared to Caucasians [34].

**Gender and host response.** Although the literature is still controversial about gender influence in C-MIP, local and systemic inflammatory biomarkers are differently expressed in males and females. Tavakoli et al. [18] reported increased GCF levels of TNFα, IFNγ, MCP1, and MIP-1α in diseased sites of males. Interestingly, healthy sites in male patients diagnosed with LAP/C-MIP also presented high levels of IFNγ and a tendency to higher G-CSF levels as well. On the other hand, peripheral blood from C-MIP females stimulated with *P. gingivalis* e *E. coli* LPS showed an increase in systemic biomarkers, such as Eotaxin, IFNγ, and GM-CSF, compared to C-MIP males [18]. Considering the age range of disease initiation in the permanent dentition, sex hormones may indeed have an impact on both the local and systemic inflammatory response. In fact, high testosterone levels in males have been correlated to periodontitis prevalence and severity [35], while estrogen reduction in a woman’s lifetime may increase the risk of periodontal issues due to its protective role [36]. However, based on the results above regarding systemic responses, it can be inferred that females diagnosed with C-MIP have a hyperresponsive tendency to biofilm challenge (LPS), which may impact faster periodontal apparatus loss [18]. Thus, hormonal imbalances during puberty in both males and females and their impact on C-MIP predisposition or initiation certainly warrant further investigation. 

**Genetic influence on host response.** Genetic aspects could also have an impact on the host inflammatory response [37,38,39]. Toll-like receptors (TLR), e.g., TLR-2 and TLR-4, have been associated with LAP/C-MIP hyper-inflammatory response [25]. This transmembrane glycoprotein pathway has shown more than a two-fold-increase in specific gene expression correlated to inflammation, such as TICAM-1 (TRIF), FOS, IRAK1, TLR2, and CCL2 in LAP/C-MIP patients compared to healthy unrelated controls [40]. IRAK1 displayed a significantly increased expression in these subjects, which may be explained by IL-1β increase in these patients [41]. Epigenetic regulation plays an important role in host immunological response via the TLR pathway. According to Shaddox et al. [42], up- (e.g., MYD88, MAP3K7, RIPK2, IL6R) and downregulation (e.g., FADD, PPARA, IRAK1BP1) of several genes can be explained by DNA methylation in specific sites. For example, methylation in FADD position 5 was positively correlated with LPS-stimulated levels of IL-6 and TNF-α systemically. Conversely, MYD88 methylation in position 4 presented a negative correlation with IL-10, IP-10, and MCP-1 [42]. 

Several gene single nucleotide polymorphisms (SNP) can influence immunological patterns and susceptibility in aggressive forms of periodontitis. IL-1α (rs1800587) and IL-1-β (rs1143634) polymorphisms have been correlated to generalized aggressive periodontitis [43]. SNP in the Lactoferrin gene, the iron-binding protein with antimicrobial activity related to host first-line defense (G-allele), was detected in young African Americans [44] and Taiwanese [45] diagnosed with aggressive forms of periodontitis. Similarly, cathepsin C SNP (rs3888798), a cysteine protease involved in neutrophil maturation and its activity in immune response, was also associated with this disease, along with lower PMN function, enhancing the risk of aggressive periodontitis development [46]. Several reviews of the literature evaluating the role of genetics in aggressive periodontitis have been published [37,47,48,49,50,51]. Given the several genes potentially associated with the disease and its inflammatory pathways, it is likely that several genetic polymorphisms are associated with susceptibility to this disease. There is also some evidence that indicates a possible genetic predisposition to specific microbial colonization [52,53]. Thus, comprehensive genomic studies with a high number of diseased individuals from different populations need to be conducted to clarify specific genes and their role in this disease. 

Finally, some studies have investigated the roles of small RNAs, known as microRNAs, or miRNAs, in periodontitis [40,54,55] and other inflammatory diseases [56]. In our cohort of LAP individuals, several miRNAs, including miR-9-5p, 155-5p, and 147a, all presented elevated expression (2-fold up-regulated) in our LAP/C-MIP cohort [40]. Interestingly, increased miR-9-5p levels were previously found in inflamed gingival tissue from patients diagnosed with periodontitis [54] and also in neutrophils and monocytes post-TLR2-4 activation [57], and both miR155-5 and 147a have also been found elevated post-LPS stimulation, promoting further inflammatory cascade [58,59,60]. There seems to be an important role for miRNA regulation in TLR responsiveness in this disease, which deserves to be further explored by comprehensive next-generation sequencing approaches at different disease stages.

## 3. Microbiological Aspects

Literature has shown a microbial profile associated with localized early-onset periodontitis [61,62,63,64,65]. Microorganisms such as *A. actinomycetemcomitans*, *T. lecithinolyticum*, and *T. forsythia* have been strongly associated with this condition [62,64]. Moreover, studies have shown that *P. gingivalis*, *P. intermedia*, *T. denticola*, *C. gracilis*, *E. nodatum*, and *F. nucleatum* are highly correlated with active disease and its prevalence [63,66]. *A. actinomycetemcomitans* has been classified as a key pathogen in young adults diagnosed with LAP/C-MIP [63,64,65,66,67,68,69] and even with sites with progressive bone loss [70,71]. This microorganism has different mechanisms of action including direct and indirect ways of influence in host response [72,73,74]. Seven serotypes can be linked with this species (a–g) based on membrane polysaccharides surface. A particular JP2 Genotype from serotype “b” has been highly associated with a severe form of periodontal disease [12,75,76] and in sites with progressive bone loss as well [76,77]. In the beginning, this species was identified in subjects from North and West Africa. Due to the transatlantic slave trade, new colonization was spread out in North and South America [75], which could explain the higher prevalence of this disease in African descendants in both these regions. In fact, JP2 clone has been reported in aggressive periodontitis in both North [78] and South American populations [67]. One of the main virulence factors from this genotype/serotype “b” is the expressive release of leukotoxin (LtxA), intensifying leukotoxic and cell death in high doses [79]. Not only does LtxA depreciate leukocyte activity, but it also increases lysosomal release in macrophages reducing its phagocytic profile [80]. 

Tissue invasion is a well-known propriety from *A. actinomycetemcomitans*, and it has already been identified in periodontal tissues from young adults [81], even at a very young age [82]. Several virulence factors in relation to this action have been described in in vitro and in vivo analysis [83]. Outer membrane protein 100 (Omp100) present *in A. actinomycetemcomitans* membrane surface composition has the capacity to bind in human epithelial cells [84]. After its adhesion, other Omp protein class groups act specifically in tissue invasion. Omp29 allows microorganism diffusion into epithelial cells, enhancing permeability due to F-actin rearrangement via FAK signaling cascade [85]. In the same way, cytolethal distending toxin (CDT) produced by this bacterium has the capacity to impact the cell cycle, leading to epithelial layer organization disruption due to the dissolution of cell junctions (e.g., cadherin) [86]. This scenario results in soft tissue collapse, allowing microorganisms and their toxins to make intimate contact with the connective tissue below [86]. 

Early *A. actinomycetemcomitans* acquisition can be correlated with C-MIP in primary and mixed dentition [64,71,75,87]. A longitudinal study reported that individuals containing *A. actinomycetemcomitans*, *S. parasanguinis*, and *F. alocis* are more prone to develop LAP/C-MIP disease [71]. In fact, a higher risk for bone loss was observed when the three microorganisms coexisted [71]. Jensen et al. have shown that healthy Moroccan toddlers between 7–10 years presented a higher CAL in mixed dentition when the JP2 genotype of *A. actinomycetemcomitans* was detected in their biofilm at incisor and molars [12]. Indeed, studies have reported a higher risk of bone loss when either non- or JP2 genotype is part of their microbiota in advance [76,77]. Aberg et al. [77] evaluated a 2-year progression of CAL based on the presence of JP2 and non-JP2 genotypes of *A. actinomycetemcomitans* in 500 adolescents (from 10 to 19 years of age). After the longitudinal follow-up, it was possible to predict a higher progression of CAL ≥ 3 mm when JP2 (OR = 14.3) and non-JP2 genotypes (OR = 3.4) were detected in subgingival biofilm. Moreover, an increasing risk of disease development followed by CAL ≥ 3 mm (RR = 7.3) was detected in healthy individuals at baseline with JP2 identification in subgingival biofilm. This evidence emphasizes the role of specific microorganisms in aggressive forms of periodontitis [77].

One of the possible explanations for this precocious colonization is family aggregation. Monteiro et al. [88] have reported a vertical pathogenic microbiome transmission between parents diagnosed with grade C periodontitis and their progeny. In fact, offspring’ subgingival biofilm from parents diagnosed with periodontal disease has shown exclusive microorganisms such as *Filifactor alocis*, *Porphyromonas gingivalis*, *Streptococcus parasanguinis*, *Fusobacterium nucleatum subsp. nucleatum* when compared to progeny from parents who do not suffer from grade C periodontitis. Moreover, even after biofilm maintenance, several periodontopathogenic species remain stable and in higher abundance in offspring from periodontal diagnosed families, including *A. actinomycetemcomitans*, *P. gingivalis*, *T. denticola*, and *T. forsythia* [88]. 

**Figure 4:**
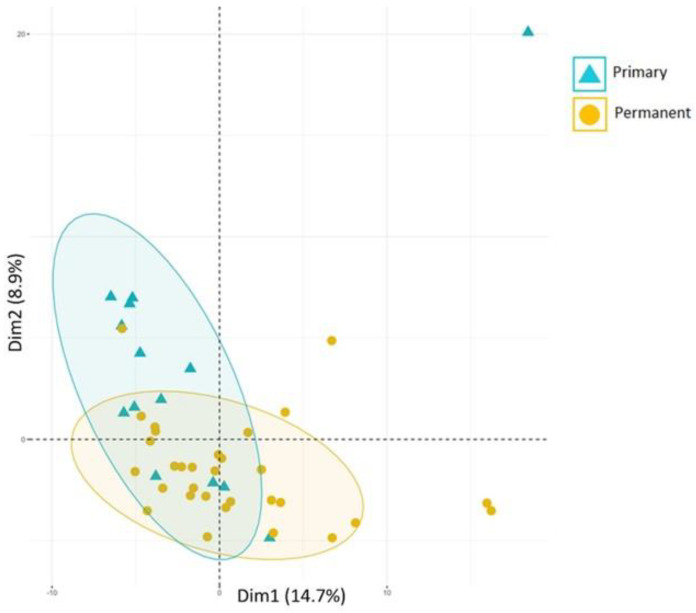
Beta-diversity analysis of C-MIP sites in children affected in the primary and permanent dentitions based on detection levels for all taxa in the HOMIM microarray. Principal component 1 (PC1, x-axis, 14.7%) vs. principal component 2 (PC2, y-axis, 8.9%) account for 23.6% of the total data variability. Ellipses represent 90% of the data variability within each group. Primary and Permanent groups differed when compared (PERMANOVA *p* < 0.01) and after adjusting for probing depth (PD) (PERMANOVA *p* < 0.05). (Source of the image [89]).

Microbiome profiles in diagnosed C-MIP subjects can distinguish between primary and permanent dentition. According to a recent study (Koo et al. [89]), a partial overlap was observed between the two types of dentitions in disease conditions with the detection of distinguished microorganisms in primary and permanent affected sites (Figure 4). *Capnocytophaga ochracea*, *Leptotrichia buccalis*, *goodfellowii*, and *Sneathia sanguinegens* were found more frequently in primary teeth-affected sites when compared to the permanent ones. On the other hand, *Filifactor alocis*, *Tannerella forsythia*, and *Synergistetes sp* were elevated at C-MIP permanent sites, whereas *A. actinomycetemcomitans*, *Campylobacter*, *Fusobacterium nucleatum*, and *Gemella morbillorum* were identified in both primary and permanent affected sites characterizing the overlapping between them. In fact, *A. actinomycetemcomitans* was highly abundant in both affected dentitions (85% and 71%, respectively), corroborating its important role in this disease, both in primary and permanent dentitions. It seems that a more mature microbiome is developed in permanent diseased sites. However, the identification of some periodontopathogens, such as *Campylobacter gracilis* and *F. nucletum ss nucleatum* were also observed early in the primary dentition [89], which has been reported by the literature in early-onset periodontitis [90,91]. Thus, despite their low abundance, primary dentition already exhibits an identification of microorganisms that potentially increase the risk of disease development in the latest detention. Moreover, the high abundance of *A. actinomycetemcomitans*, regardless of dentition, highlights this species’ role in C-MIP disease. 

An important point to consider is intrafamilial transmission and its role in the acquisition of periodontopathogens. It has been shown that individuals from the same family harbored the same biotype and serotype of *A. actinomycetemcomitans* [92], and Christersson also found that members of the same family with LAP also harbored the same biotype and serotype of *A. actinomycetemcomitans* [93]. Haubek et al. also reported the same isolates of JP2 clones in African LAP families and suggested that the strong familial aggregation of this disease was due to intrafamilial transmission of this virulent strain [94]. The most likely route of transmission is via the saliva and could be vertical (parent to child) or horizontal (partner to partner or sibling to sibling) [95,96]. Transmission theories have been reinforced by sequencing studies of specific strains [97,98,99]. However, it is possible that transient transmission does not lead to persistent colonization of the organism, as this will also depend on the host, bacterium characteristics, and the abundance of the transferred species, among others. There may also be genetic influence of colonization patterns, as the periodontal flora of identical twins has been shown to be more similar than that of fraternal ones [100,101].

## 4. Diseases Initial Time-Point

**Figure 5:**
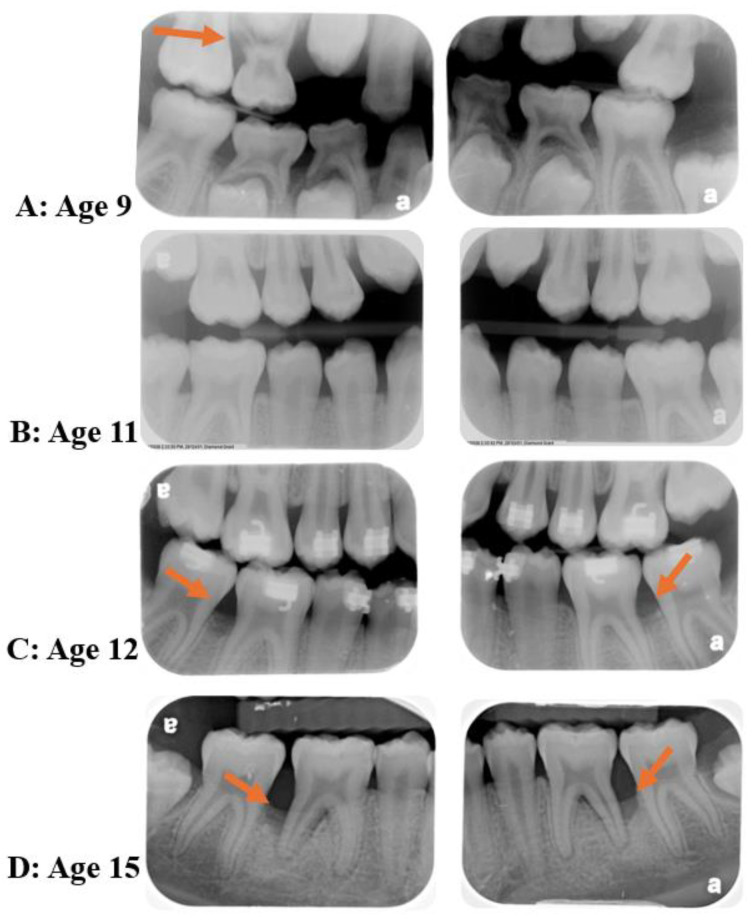
Clinical Case: The patient was referred to periodontal treatment at age 15 with significant vertical bone loss on #19 and 30. Retrospective radiographic analyses of the permanent and primary dentitions show evidence of bone loss in the upper (orange arrow) and lower primary molars at age 9 (**A**), healthy permanent dentition at age 11 (**B**), and beginning of bone defects in permanent dentition at age 12 and 15 (**C**,**D**); #19 and 30 distal surfaces) (Source of the image [10]).

**Figure 6:**
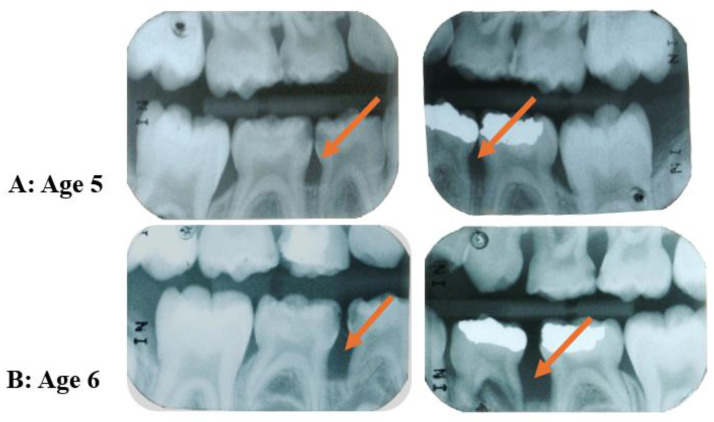
Retrospective evaluation of radiographs of a sibling of a C-MIP patient. Childhood radiographic records show initial bone loss at the first primary molars at five years old (**A**). The disease progressed fast and spread to the second primary molars at age 6 (orange arrows) (**B**). (Source of the image [16]).

**Figure 7:**
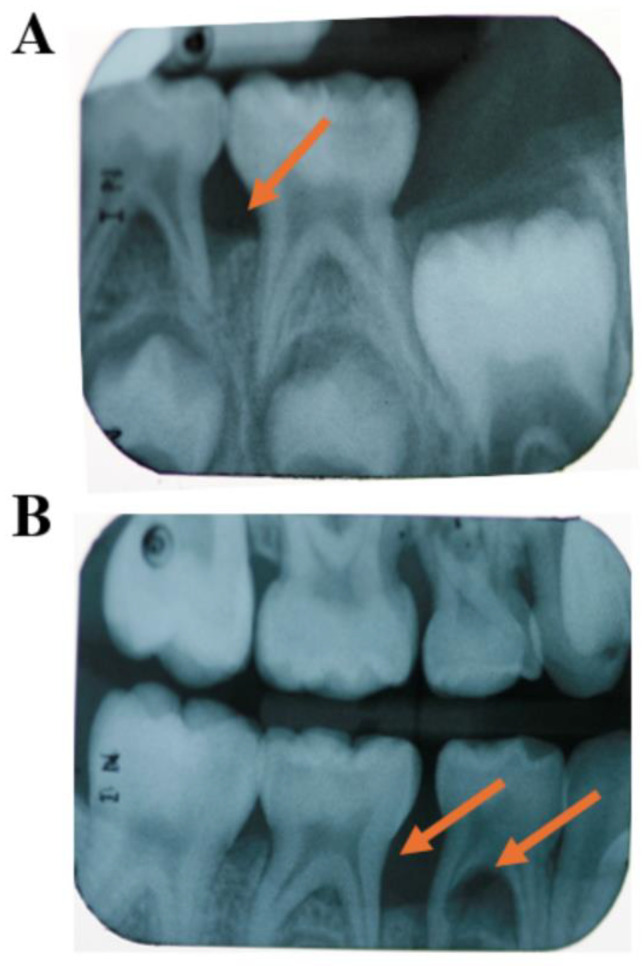
Seven-year-old African American male diagnosed with C-MIP in primary dentition. Severe bone loss on lower left primary first molar (orange arrow) (**A**). Severe bone loss of lower right first and second primary molars along with external root resorption (orange arrows) (**B**). (Source of the image [10]).

In reviewing the topics discussed above, it is possible to question whether C-MIP actually starts in the primary dentition. If left untreated, the persistent presence of the pathogen *A. actinomycetemcomitans* within tissues [93,102,103] may allow this species to recolonize the pockets around permanent dentition and within sites of hyper-inflammatory host predisposition, may lead to rapid periodontal destruction in these individuals, as studies show that treatment of this disease early in the primary dentition seems to reduce disease recurrence in the permanent dentition [15,16,17]. This microorganism can act in tissue breakdown in indirect and direct ways jeopardizing the host first-line response (PMN recruitment) all together with microorganism selectivity in the subgingival environment, respectively [72]. Other more recent retrospective analyses [10,16] were carried out evaluating radiograph bone loss patterns, external/internal root resorption, enlarged chamber pulp, and exfoliation patterns in primary dentition records from patients diagnosed with LAP/C-MIP in permanent teeth. The first study [16] had access to 39 patient’s radiographic records (20 C-MIP diagnosed and 19 healthy siblings). Ninety percent of those who had C-MIP in the permanent dentition also presented signs of radiographic bone loss around their first and second primary molars in the retrospective evaluation (Figure 5). Moreover, six of the 19 siblings who were healthy in their permanent dentition presented bone loss in primary radiographic records retrospectively, and two of them went on to develop the disease in the study follow-up [16] (Figure 6). Likewise, the second study [10] evaluated 49 periapical radiographs in 33 patients who presented the disease in the primary dentition. The first primary molar was the most affected teeth, followed by the second primary molar [10] (Figure 7). The authors suggest there seems to be a pattern of progression from the first to the second primary molar, and this could potentially lead to colonization of the first permanent molar if left untreated. Thus, it seems reasonable to infer that it is possible that C-MIP may indeed start in the primary dentition and, if left undiagnosed and untreated, may lead to disease in the permanent dentition. Thus, the role of pediatric dentists in the diagnosis and management of this disease early becomes essential for the proper treatment of this disease and prevention of possible breakdown of the permanent dentition [104]. The Academy of Pediatric Dentistry’s latest recommendation for the diagnosis of periodontal disease in children is to perform a full mouth periodontal examination as soon as the first permanent molar erupts, around 6 years of age [105], or earlier if clinical signs of disease or radiographic bone loss are detected or suspected.

## 5. Treatment Aspects

**Figure 8:**
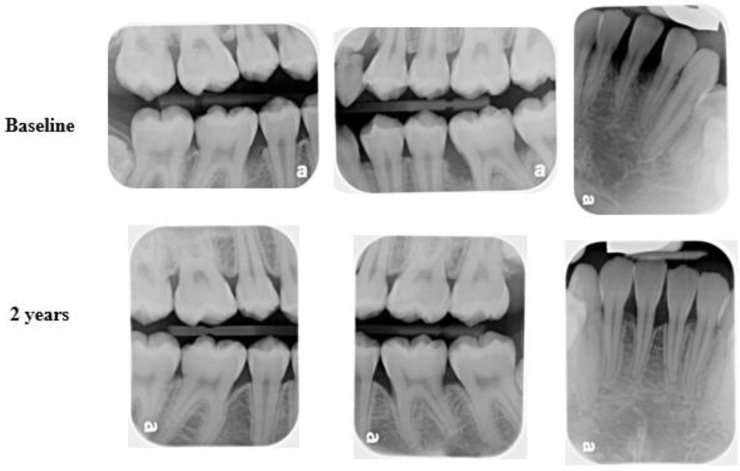
Female patient diagnosed with C-MIP presenting bone loss in the incisors (25) and 1st permanent molars (baseline-upper). After SRP + ABX treatment, bone fill and stability were achieved at 2 years of follow-up (bottom).

Scaling and root planning (SRP) associated with systemic antibiotic therapy (Amoxicillin and Metronidazole) is the current gold standard for treating C-MIP [62,106,107,108,109]. According to most clinical studies, this therapy can provide significant probing depth (PD) reduction, clinical attachment level (CAL) gain, along with positive modulation in host proinflammatory profile (GCF), maintaining microbial compatibility with health sites in the short and long term (Figure 8) [62,106,107,108,109]. However, it is important to highlight that although patients respond well to this therapy, patients who present a high LPS responsiveness may not achieve equal outcomes to the ones presenting a lower responsiveness, even under the same clinical approach [110]. 

Branco de Almeida et al. [109] and Miller et al. [16] showed great PD reduction and clinical attachment gain in diagnosed LAP/C-MIP patients when a regimen of full mouth debridement associated with systemic antibiotic therapy (ABX at baseline: Metronidazole-250 mg and Amoxicillin-500 mg, 3× per day for 7 days) immediately after the clinical debridement was the treatment of choice. Positive clinical parameter reductions were observed in the short-term (6 months) and maintained in the long-term (2 years), while Miller et al. [16] showed this reduction in clinical parameters was maintained for up to 4 years post-treatment. Moreover, several biomarkers in diseased and health sites from LAP/C-MIP patients were reduced in at least one time point when compared to baseline levels [109]. Most of these decreased biomarkers have been characterized as disease features in discriminative analysis [21]. Thus, it seems that this type of approach can lead to clinical stability and local host modulation in the long term. Despite the positive and sustained modulation in GCF biomarkers after the non-surgical periodontal therapy combined with ABX, serum biomarkers modulation does not provide the same outcome [111]. Limited cytokines/chemokines are reduced in some post-treatment time points, while others seem to rebound, especially after 6 months [112]. In fact, some studies have shown that serum inflammatory biomarkers persist at higher rates compared to diagnosed patients even after treatment [113] or return to their baseline levels after long-term follow-up [114]. Comparing C-MIP and GAgP response after 1-year long-term follow under the ABX approach, it is possible to observe similarities between both disease types of outcomes regarding serum and GCF levels [23].

**Figure 9:**
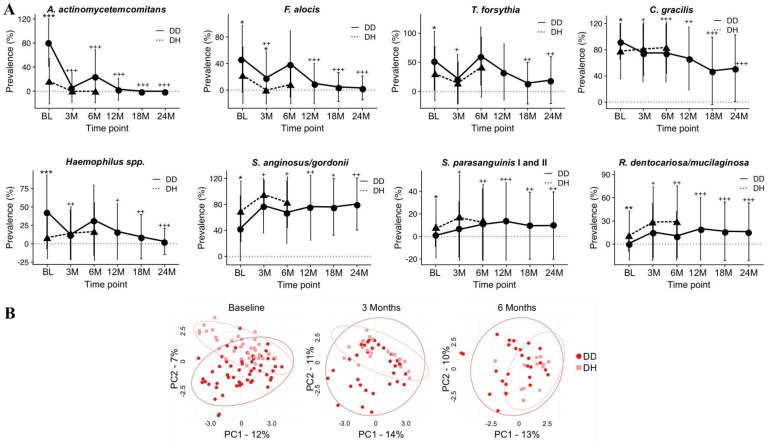
Prevalence of disease- and healthy-associated species after treatment (mechanical debridement with systemic antibiotics −metronidazole 250 mg + amoxicillin 500 mg per 7 days) of C/MIP patients over time (**A**). Principal coordinates analysis (PCoA) shows visibly separated clusters at baseline disease (DD) and healthy (DH) sites than after treatment with an overlap in bacterial profiles of DD and DH sites at 6 months (*p* < 0.05). Ellipses show 95% confidence intervals (**B**)**.** Values are mean ± SD. * *p* < 0.05, ** *p* < 0.01, *** *p* < 0.001 between DD and DH. + *p* < 0.05, ++ *p* < 0.01, +++ *p* < 0.001 in DD compared to baseline. (Source of the image [62]).

From a microbiological perspective, the same clinical approach (SRP+ABX) can modify host microbiota, reducing putative species correlated to C-MIP disease [114]. Velsko et al. [62] have shown that this protocol (debridement with a 7-day course of ABX) maintained a healthy microbial environment in a 2-year long-term, decreasing *A. actinomycetemcomitans*, *F. alocis*, *T. forsythia*, and *C. gracilis* while health-associated species, such *S. anginosus/gordonii* and *S. parasanguinis*, increased (Figure 9A). A statistical difference in community profile could be observed between healthy and diseased sites before therapy, and a closer cluster (more similar profile) between health and disease profiles could be observed in C-MIP patients post-treatment compared to baseline (Figure 9B) [62]. Burgess et al. [78] also reported a drastic reduction in the initially high prevalence of JP2 genotype post-treatment, and this significant reduction was maintained for 12 months post-treatment, with one course of ABX at baseline and proper periodontal maintenance. In diseased sites, JP2 genotype prevalence was reduced to 3.23% (1/31 site detection) after 1 year of treatment compared to baseline, while no detection was observed in the health sites of African American patients diagnosed with LAP/C-MIP [78]. A different regiment of ABX (Metronidazole-400 mg and Amoxicilin-500 mg, 3× per day for 14 days) along with SRP also decreased JP2 genotype in LAP/C-MIP patients after 1 year. Additionally, serum IgG against Omp29 was also decreased, which is one of the virulence factors correlated to tissue invasion [106].

Considering dentition type under SRP+ABX clinical approach, Merchant et al. [9] have shown a better clinical attachment gain in primary dentition at 3, 6, and 12 months compared to the permanent dentition, although both dentitions presented a significant reduction in all clinical parameters post-treatment. These results were encouraging regarding early treatment of disease and indicated that younger patients may be more likely to have disease resolution [9]. This can be associated with a few conditions, such as low inflammatory rates in younger children [115], as well as precocious disease diagnosis and intervention under a less complex dysbiotic environment [89]. Depending on the amount of bone level compromised due to disease progression, primary tooth extraction may be an alternative, especially in cases with extensive mobility, pain/abscess, or loss of function impact on quality of life [116]. 

There is no study confirming disease prevention in permanent teeth in patients diagnosed and treated in the primary dentition. However, the few longitudinal studies following patients diagnosed with LAP/C-MIP in primary dentition indicate a favorable response to treatment and a low incidence of disease in permanent dentition with proper maintenance. According to Mros and Berglundh [15], no bone loss was detected in 7 out of 13 subjects previously diagnosed with the disease in early dentition under proper periodontal maintenance. Miller et al. did not report a recurrence of the disease in permanent dentition in a long-term follow-up of the Florida cohort [16]. Despite the small sample size, low rates of disease recurrence were also observed by Bimstein [17] and Merchant et al. [9]. Thus, it seems reasonable to reinforce periodontal screening in pediatric dental appointments focusing on early diagnosis, especially when cases of aggressive periodontitis are reported in the family.

## 6. Conclusions

C-MIP is an oral disease more prevalent in African descendants and young systemically healthy adults (under 35 years old) that affects the tooth-supporting apparatus of very specific teeth and features rapid alveolar bone loss. Most cases are associated with low biofilm accumulation, bone loss affecting first molars and incisors, along with familial aggregation. Although less studied, this disease can also be diagnosed in the primary dentition, which may lead to more successful treatment and possibly prevent the occurrence of the disease in the permanent dentition. Studies evaluating primary dentition records have shown that most patients diagnosed in the permanent dentition had disease signs (i.e., bone loss) in the primary dentition. 

A heightened inflammatory response to bacteria can be observed in these patients, and healthy siblings also present a tendency of high inflammatory response even without a clinical diagnosis, which may be a result of a genetic predisposition to the disease, given the similar patterns of host response seen in families. The immunological response to biofilm is high regardless of the stimuli used. Periodontal therapy has been shown to successfully manage the local inflammatory profile and clinical response to treatment; however, the modulation of serum biomarkers is not as consistent, and some markers seem to remain high or rebound in the long term despite clinical response remaining positive. 

*A. actinomycetemcomitans* is a key pathogen either in primary or permanent dentition affected with this disease, characterized by special tools for tissue invasion and more virulent genotypes (e.g., JP2). *A. actinomycetemcomitans* early acquisition was also reported as a possible predictor to disease initiation. The colonization of *A. actinomycetemcomitans* in primary dentition can be explained by familial aggregation and possible pathogenic transmission within families. Similar subgingival biofilm composition between parents diagnosed with an aggressive type of periodontitis and their offspring has been reported in the literature. In fact, the bacterial community in these children seems to be resistant even after periodontal maintenance. After periodontal therapy, periodontopathogens are significantly reduced, and this profile can be maintained for a long period with proper supportive periodontal therapy. 

SRP + ABX is the gold standard for treating C-MIP, supported by several studies and systematic reviews. Most studies report amoxicillin and metronidazole as the regimen of choice as adjuncts to SRP/full mouth debridement. The disease affecting both primary and permanent dentition presents a favorable clinical response to treatment. However, higher gains in clinical attachment levels seem to happen in younger children. This may be explained by a lower inflammatory profile in young children [115] and a less mature microbial community compared to permanent dentition [89]

## 7. Future Directions

There are still many gaps to be investigated in C-MIP, such as sex hormones (androgens, estrogens, and progestogens) and other cofactors during childhood development that may influence the disease incidence. Moreover, epigenetic studies and other comprehensive analyses using integrative -omics analysis (e.g., proteomic, metagenomic, genomics) should be carried out focusing on better understanding the biomolecular features of this disease in different time points and in different populations. In addition, there is not yet a group of feature inflammatory biomarkers to control diagnosis, progression, and disease severity over time. A better understanding and correlation between serum, GCF, and saliva biomarkers may guide research and clinical practitioners to a more assertive approach to this disease. Despite the great success under SRP+ABX therapy, host modulation, e.g., pro-resolving lipid mediators, may be an alternative option in future studies [117,118,119], given the increase in ABX resistance and the heightened inflammatory response seen in these individuals. Considering the current classification system, it is important to include clinical characteristics that have been discussed and published in the past for this disease in different stages so that studies with specific inclusion criteria are conducted, and knowledge continues to be developed for this particular disease in the future. Moreover, given the fact that some evidence indicates a possible disease initiation in the primary dentition, periodontal screening during pediatric appointments remains crucial for early disease diagnosis and treatment and possible prevention of disease at later stages. 

## Data Availability

Not applicable.
